# Attributed graph distance measure for automatic detection of attention deficit hyperactive disordered subjects

**DOI:** 10.3389/fncir.2014.00064

**Published:** 2014-06-16

**Authors:** Soumyabrata Dey, A. Ravishankar Rao, Mubarak Shah

**Affiliations:** ^1^Department of Electrical Engineering and Computer Science, Center for Research in Computer Vision, University of Central FloridaOrlando, FL, USA; ^2^Self, Consultant, Data ScienceYorktown Heights, USA

**Keywords:** attention deficit hyperactive disorder, functional magnetic resonance imaging, support vector machine, multidimensional scaling, attributed graph

## Abstract

Attention Deficit Hyperactive Disorder (ADHD) is getting a lot of attention recently for two reasons. First, it is one of the most commonly found childhood disorders and second, the root cause of the problem is still unknown. Functional Magnetic Resonance Imaging (fMRI) data has become a popular tool for the analysis of ADHD, which is the focus of our current research. In this paper we propose a novel framework for the automatic classification of the ADHD subjects using their resting state fMRI (rs-fMRI) data of the brain. We construct brain functional connectivity networks for all the subjects. The nodes of the network are constructed with clusters of highly active voxels and edges between any pair of nodes represent the correlations between their average fMRI time series. The activity level of the voxels are measured based on the average power of their corresponding fMRI time-series. For each node of the networks, a local descriptor comprising of a set of attributes of the node is computed. Next, the Multi-Dimensional Scaling (MDS) technique is used to project all the subjects from the unknown graph-space to a low dimensional space based on their inter-graph distance measures. Finally, the Support Vector Machine (SVM) classifier is used on the low dimensional projected space for automatic classification of the ADHD subjects. Exhaustive experimental validation of the proposed method is performed using the data set released for the ADHD-200 competition. Our method shows promise as we achieve impressive classification accuracies on the training (70.49%) and test data sets (73.55%). Our results reveal that the detection rates are higher when classification is performed separately on the male and female groups of subjects.

## 1. Introduction

Attention Deficit Hyperactive Disorder (ADHD) is one of the most commonly found functional disorders affecting children. Around 5–10% of school aged children are diagnosed with ADHD (Biederman, 2005). In spite of all the efforts made in the studies of ADHD, the root cause of this problem is still unknown. No well known biological measure exists to date to detect ADHD. Instead, it is characterized by clinical symptoms such as inattention, impulsivity and hyperactivity all of which are subjective. In the proposed method we try to address the problem of automatic classification of the ADHD subjects from their rs-fMRI data alone. For this purpose we construct the resting state functional connectivity network of the brain and exploit the topological differences of the networks of the ADHD and control subjects for classifications. In the rest of the article, the words network and graph are used interchangeably with similar meaning.

Recently, fMRI has become very popular for brain activity related studies. Researchers use it for identifying the brain regions which are responsible for particular cognitive activities based on the correlation of input stimulus signal and captured brain fMRI signals (task-related fMRI). Also, it is used for better understanding of different brain functional diseases like dementia (Rombouts et al., 2009). Likewise, ADHD is also being studied under the light of structural and functional brain imaging techniques. Structural MRI (sMRI) analysis suggests that there are abnormalities in ADHD brains, specifically in the frontal lobes, basal ganglia, parietal lobe, occipital lobe, and cerebellum (Castellanos et al., 1996; Overmeyer et al., 2001; Sowell et al., 2003; Seidman et al., 2006). In another set of studies ADHD brains were analyzed using task-related fMRI data. Bush et al. (1999) found significant low activity in the anterior cingulate cortex when ADHD subjects were asked to perform the Counting Stroop during fMRI. Durston (2003) showed that the ADHD conditioned children have difficulties performing the go/nogo task and display decreased activity in the frontostriatal regions. Teicher et al. (2000) demonstrated that the boys with ADHD have higher T2 relaxation time in the putamen which is directly connected to a child's capacity to sit still. A third set of works was done using the resting state brain fMRI to locate any abnormalities in the Default Mode Network (DMN). Castellanos et al. (2008) performed Generalized Linear Model based regression analysis on the whole brain with respect to three frontal foci of DMN and found low negative correlated activity in precuneus/anterior cingulate cortex in ADHD subjects. Tian et al. (2006) found functional abnormalities in the dorsal anterior cingulate cortex, (Cao et al., 2006) showed decreased regional homogeneity in the frontal-striatal-cerebellar circuits but increased regional homogeneity in the occipital cortex among boys with ADHD, (Zang et al., 2007) verified decreased Amplitude of Low-Frequency Fluctuation (ALFF) in the right inferior frontal cortex, left sensorimotor cortex, bilateral cerebellum, and the vermis, as well as increased ALFF in the right anterior cingulate cortex, left sensorimotor cortex, and bilateral brainstem.

While studies of group level statistics may indicate the abnormal regions of ADHD patients, their use for automatic diagnosis is still under investigation. There have been relatively few investigations at the individual level of classification of the ADHD subjects. One such study was performed by Zhu et al. (2008) where ADHD subjects were classified based on the regional homogeneity of their fMRI data. In another work, bag-of-words framework was used by Solmaz et al. (2012) for the classification of the ADHD subjects. Recently, there was a global competition (ADHD-200) organized, involving researchers from different scientific disciplines, for automatic diagnosis of ADHD subjects as well as understanding the underlying pathophysiology. For this purpose the organizers released a large data-set containing rs-fMRI data, sMRI data and phenotypic information of ADHD and control subjects. Different automatic classification methods were published using this data-set (Bohland et al., 2012; Brown et al., 2012; Chang et al., 2012; Cheng et al., 2012; Colby et al., 2012; Dai et al., 2012; Dey et al., 2012; Eloyan et al., 2012; Olivetti et al., 2012; Sato et al., 2012; Sidhu et al., 2012). Many of these approaches used some combination of rs-fMRI, sMRI and phenotypic data. Cortical thickness, gray matter probability, texture of structural brain images were some of the common sMRI features used for the classification. Regional homogeneity, and Fourier transformation of fMRI signal were some of the features used from functional images. Several studies computed functional networks from fMRI data and used different network statistics as features. Brown et al. (2012) showed that even the use of only phenotypic features can produce high classification accuracy. All of these works achieved classification accuracy higher than the chance factor.

**Figure 1:**
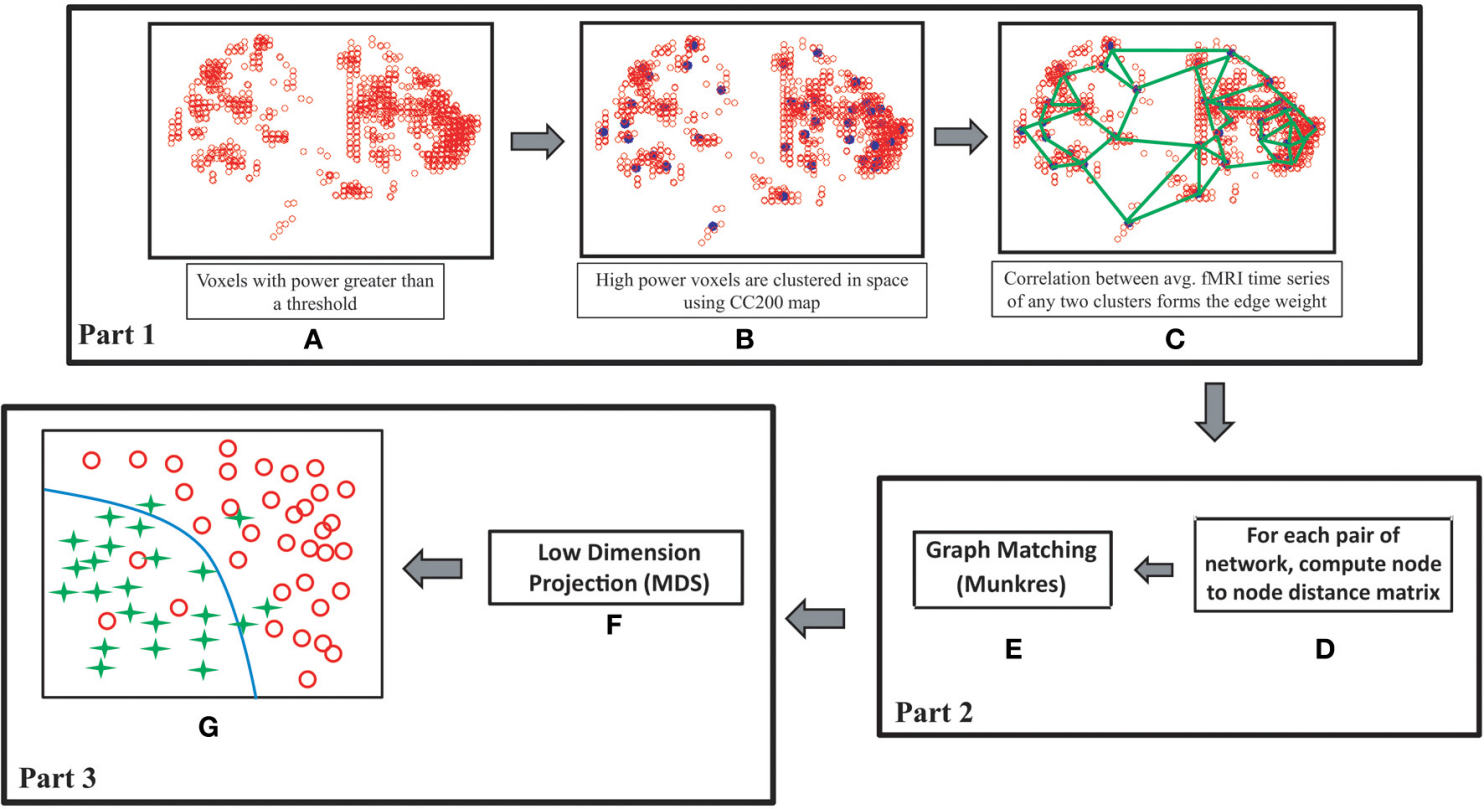
**Flowchart of our proposed method. (A)** High power voxels are selected, **(B)** voxels belong to each region of interest of CC200 map are clustered together and represented by their cluster centers, **(C)** edges of the network are formed based on the correlations of average fMRI signals of the clusters, **(D,E)** inter network distances are computed in two steps. First, for a pair of networks a node to node distance matrix is computed. Next, each node of the network with fewer node count is assigned to a node of the second network using Munkres algorithm such that total matching distance is minimized. **(F)** MDS is used to form a spatial configuration of the subjects on a low dimensional space based on the inter-graph distance measures, **(G)** classification is performed in the projected space.

As discussed, researchers have identified considerable differences between the ADHD and control groups while analyzing rs-fMRI data. This motivates us to use the rs-fMRI data of the ADHD-200 competition data set for the validation of our proposed classification algorithm. Use of data from other modalities like structural MRI and phenotypic information might improve the classification accuracies but our aim is to verify the effectiveness of rs-fMRI data only for solving the proposed problem. As shown in Figure 1, our method can be subdivided into three main parts. In the first part we construct the resting state brain functional connectivity networks for the subjects under consideration. The networks are modeled as attributed graphs where each node is assigned a signature. Attributed graphs are used previously in different works (Jouili and Tabbone, 2009; Xu et al., 2012). The signature of a node is a set of attributes which characterizes the node. The attribute set includes the degree of the node, the degree of the neighboring nodes, the power of the node, the power of the neighboring nodes and the physical location of the node. The power of a node is calculated by averaging the power of the fMRI time series of all the voxels comprising the node. In the second part we compute distances between all possible pairs of graphs. The distance computation for a pair of graphs is a two step process. In the first step distances for all the node pairs are computed based on their signature values. In the next step, all nodes of one graph are assigned to the nodes of the second graph such that the total matching cost is minimized. The Munkres algorithm is used for the node assignment problem (Munkres, 1957). In the last part the graphs are projected to a space of specified dimensions based on their distance measures. The MDS (Torgerson, 1952) method is used for this purpose. Finally, a Support Vector Machine (SVM) is used for the classification of ADHD subjects in the projected space. The main contribution of our work is to propose a novel automatic classification framework of ADHD subjects based on the topological differences of the functional brain connectivity networks of the ADHD and control groups of subjects. Unlike the other methods, which use functional brain networks for ADHD subject classification, we refrain from using network features. Instead we mapped the networks onto a low dimensional spatial configuration and perform classification on the projected space. We also provided physical interpretations of each of the dimensions of the projected space. We achieve impressive detection accuracies on training (70.49%) and test sets (73.55%). To the best of our knowledge, our average detection rate on the test sets outperforms the previous best results (69.59% by Dey et al., 2012).

The rest of the article is organized as follows. Data descriptions are provided in section 2.1. In section 2.2, we provide brief introduction of MDS. The main method is described in 2.3.1, 2.3.2, and 2.3.3 sections.

## 2. Materials and methods

### 2.1. Data

The data, provided by Neuro Bureau for the ADHD 200 competition, is used for our study. Eight different centers contributed to the compilation of the whole data set, which makes it diverse as well as complex. In total it consists of 776 training and 197 test subjects. Different phenotypic information, such as age, gender, handedness, IQ, is also provided for each subject. The experimental validations of our proposed method are performed on the training and test data sets of 4 of the data centers - Kennedy Krieger Institute (KKI), Neuro Image Sample (NeuroImage), Oregon Health and Science University (OHSU) and Peking University (Peking). Also, based on the information provided with the phenotypic data, we excluded all those subjects from our study which have questionable functional image quality (*QC_R_est*_1_ = 0 of the phenotypic data sheet). Consider Table 1 for an overview of the data used in our study. Different data centers used different scanners and scanning parameters for capturing data. For example KKI and NeuroIMAGE used Siemens Trio 3-tesla scanner, OHSU used Siemens Magnetom TrioTim syngo MR B17 scanner and Peking used Siemens Magnetom TrioTim syngo MR B15 scanner. Some important scanning parameters used by the data centers are listed in Table 2. Also different data acquisition parameters are used by different data centers such as KKI and NeuroIMAGE captured data with subjects' eyes closed, OHSU and Peking asked their subjects to keep their eyes open. While OHSU showed a fixation cross at the screen, Peking didn't show anything. All research conducted by ADHD-200 data contributing sites were performed with local IRB approval, and contributed in compliance with local IRB protocols. In compliance with the Health Insurance Portability and Accountability Act(HIPAA) privacy rules, all data used for the experiments of this article are fully anonymized. The competition organizers made sure that the 18 patient identifiers as well as face information are removed.

**Table 1:** **Summary of the training and test data-sets from four test centers which are used in our work**.

**Center**	**Sub Cnt**	**Age (years)**	**Male**	**Female**	**Control**	**Combined**	**Hyperactive**	**Inattentive**
**TRAINING DATA-SET**								
Kennedy Krieger Institute	78	8–13	42	36	57	16	1	4
Neuro image sample	39	11–22	25	14	22	11	6	0
Oregon Health and Sci. Univ.	66	7–12	34	32	38	15	1	12
Peking University	183	8–17	135	48	114	22	0	47
**TEST DATA-SET**								
Kennedy Krieger Institute	11	8–12	10	1	8	3	0	0
Neuro image sample	25	13–26	12	13	14	11	0	0
Oregon Health and Sci. Univ.	34	7–12	17	17	27	5	1	1
Peking University	51	8–15	32	19	27	9	1	14

The data was released for ADHD-200 global competition.

**Table 2:** **Table lists the summary of scan parameters for all the data centers**.

	**KKI**	**NeuroIMAGE**	**OHSU**	**Peking**
*TR*/*TE* (ms)	2500/30	1960/40	2500/30	2000/30
Slices	47	37	36	33
Thickness (mm)	3.0	3.0	3.8	3.5
FoV read (mm)	256	224	240	200
FoV phase (%)	100	100	100	100
Flip angel (degree)	75	80	90	90

For all our experiments we used the preprocessed rs-fMRI data released for the competition. The preprocessing is performed by the competition organizers using the AFNI Cox (1996) and FSL Jenkinson et al. (2012) tools and computed on Athena computer clusters at the Virginia Tech advance research computing center. All the fMRI scans are slice timing corrected, motion corrected to the first image of the time series, registered on a 4 × 4 × 4mm voxel resolution Montreal Neurological Institute (MNI) space, filtered using a bandpass filter (0.009 Hz <f <0.08 Hz), and blurred with a 6-mm FWHM Gaussian filter. We used a binary mask, provided with each of the subjects, to find out the voxels which are inside the brain volume. All the fMRI data volumes are of size 49 × 58 × 47 voxels, but the number of samples across time varies among the data capturing centers. For further information about the data and preprocessing steps and how to access the freely available data we refer the interested readers to the following web document (NITRC, 2011).

### 2.2. Multidimensional scaling

We provide a general overview of MDS for the sake of the completeness of the paper. MDS is a set of data analysis techniques that enables one to understand the key dimensions of the objects under investigation. The method and term were first introduce by Torgerson (1952). Given a set of objects and the proximities of each possible pairs of objects, MDS techniques can find a spatial configuration of the objects based on their proximities. Here, proximities suggest the overall dissimilarities or similarities of the objects being considered. Hence, MDS can be viewed as a method to project the objects from a space of unknown dimensions to a space of specified dimensions such a way that the original proximities of the objects are preserved as closely as possible. To state it formally, given *N* numbers of objects and a dissimilarity (or similarity) matrix *D_NxN_*, MDS projects the objects on a space of given dimensions in such a way that *D* − *D_p_* is minimized. *D_p_* is the distance matrix in the projected space.

Depending on how a dissimilarity (or similarity) matrix is computed, MDS can be subdivided into direct and indirect methods. While for the direct methods numerical dissimilarity value of each pair of objects can be directly computed, for the indirect methods the dissimilarity values need to be derived from other values like confusion data. MDS can be divided into classical and nonmetric classes depending on how the problem is solved. While the classical methods assume that the dissimilarity matrix contains exact distances of the objects, the nonmetric methods consider only the ordinal information of the object proximities. For more details on the MDS we refer the interested readers to Kruskal and Wish (1978). For our experiments we used a direct classical MDS technique.

### 2.3. Method

The proposed method can be divided into three main parts such as network construction, graph distance computation and ADHD subject classification. The following sections describe each of the parts in details.

#### 2.3.1. Network construction

For all the subjects of the data set the resting state functional connectivity networks are computed. The following steps describe the network construction method and the concept is graphically explained in Figures 1A–C.

The first step of the network construction method is the selection of the candidate voxels which constitute the network. We observe that all the brain voxels do not contain valuable information and including irrelevant voxels can degrade the classification performance. This motivates us to select the voxels with high activity level which are more effective in modeling the functional connectivity networks and also in discriminating the ADHD and the control groups of subjects. We substantiate our observation by examining experimental data in Section 3, where we show that the inclusion of all the brain voxels in the construction of the network degrades classification performance. We consider the power of the fMRI time series of a voxel as the measure of its activity. The higher the power of a voxel, the higher is its activity level. For a discrete time series *T* = {*t*_1_, *t*_2_, …, *t_n_*}, the power can be computed as,

(1) $$P(T)=\frac{1}{n}\sum_{i=1}^{n}t_{i}{}^{2}$$

We then normalize the power values of all voxels between [0, 1]. The voxels are then ranked based on their power values. Finally, for the network construction we select the voxels ranked with 98 percentile or more.

The second step of the network construction method is to decide how to represent the nodes of the network. One easy solution is to assign every voxel to a node of the network. The problem of doing this is that it makes the size of the network very large, which is inefficient for further computational analysis. Also, the network constructed in this fashion is full of redundant information as the voxels in close spatial proximity have very similar functional activity patterns. For these reasons we use a functional regions of interests (ROIs) map, (CC200) proposed by Craddock et al. (2011), to construct the nodes of the network. The map is generated by parcellating whole brain resting state fMRI data into 190 spatially coherent regions of homogeneous functional connectivity (FC). We cluster all the selected voxels belong to the same ROIs and represent each of the clusters as a node of the network. The issue concerning the best resolution of ROIs which contains maximum information with minimum redundancy for the functional study of the brains is not addressed in this work.

In the third step we construct the edges of the network and compute the weights of the edges. We represent each node by the average fMRI time series of all the voxels comprising the node. Then, a correlation matrix is computed which contains correlation values of the fMRI time series of all possible pairs of the nodes in the network. For two nodes *m* and *n* with fMRI time series *m_T_* = {*m*_1_, *m*_2_, …, *m_t_*} and *n*_T_ = {*n*_1_, *n*_2_, …, *n_t_*} respectively, the correlation value is computed as:

(2) $$corr(m_{T},\;n_{T})=\frac{\left(t\sum_{i=1}^{t}m_{i}n_{i}\right)-\left(\sum_{i=1}^{t}m_{i}\right)\left(\sum_{i=1}^{t}n_{i}\right)}{\sqrt{\left[t\sum_{i=1}^{t}m_{i}^{2}-{\left(\sum_{i=1}^{t}m_{i}\right)}^{2}\right]\left[t\sum_{i=1}^{t}n_{i}^{2}-{\left(\sum_{i=1}^{t}n_{i}\right)}^{2}\right]}},$$

Note that the correlation values have range [−1, 1]. We empirically verified that the networks constructed with only positive correlation values generate better classification accuracies than the networks constructed with only negative correlation values or absolute correlation values. Hence, the experimental results reported use the networks with edges constructed with positive correlation values only. Also, we use a correlation threshold *corrTh* to remove all the edges from the network which have correlation values less than the threshold.

In the final step, we represent the network as an attributed graph where each node of the network is represented by a set of attributes. We call it the signature of a node. Given a node *n*, its signature is defined as:

(3) Signature(n)=〈deg(n),deg(ngh(n)),pow(n),pow(ngh(n)),coord(n)〉,

where the functions, *deg*(.), *ngh*(.), *pow*(.), return sum of weights of all the edges connected, the nodes connected by an edge and the power of the input node respectively. *coord*(.) is the mean physical coordinate of all the voxels comprising the node.

#### 2.3.2. Graph distance

Once the functional networks are constructed for all of the subjects in the data set, we compute the distances of all possible pairs of networks as shown in Figure 1D. For a pair of networks distance computation is a two step process. In the first step we compute the distances of all the node pairs formed by selecting one node from each of the networks. Given two networks *G*_1_ = (*V*_1_, *E*_1_) and *G*_2_ = (*V*_2_, *E*_2_) and two nodes *v*_1_ ∊ *V*_1_ and *v*_2_ ∊ *V*_2_, the distance between *v*_1_ and *v*_2_ is computed as the difference of their signatures:

(4) $$dist(v_{1},v_{2})=W\;\cdot \;{\left[d_{1},d_{2},d_{3},d_{4},d_{5}\right]}^{T},$$

where **W** = [0.2, 0.1, 0.2, 0.1, 0.4] is the weight vector and *d*_1_, *d*_2_, *d*_3_, *d*_4_, *d*_5_ are the differences of the node degrees, the neighbor node degrees, the node powers, the neighbor node powers, and the physical locations of *v*_1_ and *v*_2_. All the difference values are normalized between [0, 1] to enable proper comparison. The values for *d*_1_ and *d*_3_ are simply calculated by computing degree and power differences of *v*_1_ and *v*_2_ and dividing them respectively by the maximum degree and power encountered for any of the nodes in the training set. To compute *d*_2_ first we sort the neighbor degrees in descending orders. The node with less number of neighbor nodes is zero padded at the end to make the size of the degree arrays same. Finally, we sum up the absolute differences of the array elements and divide the summed up value by (maximum degree * size(degree array)). *d*_4_ is computed in a similar fashion while power values are used instead of degrees. *d*_5_ is calculated as follows:

(5) $$d_{5}=\frac{1}{1\text{ + }300e^{\left(\left|c_{1}-c_{2}\right|\right)/4}},$$

where *c*_1_ and *c*_2_ are the physical coordinates of *v*_1_ and *v*_2_ respectively. This is a sigmoid curve which restricts the value of *d*_5_ to the range [0, 1]. The parameters of the equation are intuitively determined in such a manner that the value of *d*_5_ is close to zero when |*c*_1_ − *c*_2_| = 0, low for the nodes in spatial locality and steeply increasing for the nodes which are further apart. The components of the weight vector *W* are determined intuitively considering the following criteria. First, we want to make sure that the nodes which are physically far apart should not match and therefore set the highest weight corresponding to the physical distances of the nodes. Next, we want to give the same importance to the degree and power distances of the nodes. Hence, the weights corresponding to the node degrees and power distances are assigned the same value so are the neighbor node degree and power distances. Finally, we assume that the importance of the node feature distances will be higher than the importance of the neighbor nodes feature distances and hence weight for the neighbor node distances are lower than the node distances. In general the distance of a pair of graphs should be calculated in such a way that the nodes from the nearby regions with similar degrees and powers and with similar neighbor nodes' degree and power distributions should match.

In the next step, we use the Munkres assignment algorithm Munkres (1957) to assign all the nodes of one network to the nodes of second network such a way that the total assignment cost is minimized. This assignment cost is considered as distance of the network pair. Note that the numbers of nodes for all the networks are not same. This is because when we select the high power voxels there are some ROIs from which no voxels are selected.

#### 2.3.3. Classification

When the subjects are modeled as graphs, they cannot be directly used for classification but need to be mapped onto a feature space. A common way to deal with this is to compute different network features which can be used for the classification (Zhu et al., 2008; Bohland et al., 2012; Dey et al., 2012). We took a different approach to solve this problem. As shown in Figures 1E,F, we use the direct classical MDS technique to project the networks in a space with specified dimensions. The MDS method takes the network distance matrix, computed in the previous part of our method, as input and produces a spatial configuration of the networks in the projected space. The number of dimensions of the projected space can also be specified in the MDS method. We got the best classification performances when we use number of dimensions as 2. All the results of our proposed method are generated on the 2 dimensional projected space.

The classification is performed in the projected space using the SVM Cortes and Vapnik (1995) with a polynomial kernel. We choose to use the SVM classifiers for the following reasons. First, the SVM can classify the data points from two classes, which are not easily separable in the feature space, by using a kernel trick to project the data points into a hyperspace where the separation is easy. Second, the SVM regresses the feature space without over fitting on the data by allowing miss classification with a penalty. Experimental results show that the classifiers perform better when trained separately on the male and female subjects. This indicates that there may be considerable differences in the functional connectivity networks of the male and female subject groups. Our result is in concordance of the work of Bálint et al. (2009) who showed that the male and female ADHD subjects have different levels of functioning.

#### 2.3.4. Experimental setup

The setups for all the different experiments performed are described in this section. Experiment results are listed in section 3.

For all our experiments we used MATLAB (version R2008b) implementations of the MDS and SVM. For the MDS, we used the function name *mdsscale* with the *criterion metricstress* and *MaxIter* = 100000. For the SVM, we used the functions named *svmtrain* (with polynomial kernel) and *svmpredict* to train the classifiers and test the detection accuracies respectively.

For all the training and test sets of all the data centers, three different sets of experiments are performed. While the first set of experiments is performed on all the subjects, the second and third sets of experiments are performed on the male and female groups separately. Please note that the classifiers are trained separately on the training and test sets of each data center. Hence, in total [ (4 training sets + 4 test sets) * 3 ] 24 different sets of experiments are performed. For the training sets, detection accuracies are achieved by leave one out cross validation method. For the test sets, the classifiers are trained on the subjects of the corresponding training sets and detections are performed on the test sets. For each of these sets of experiments we construct the networks by varying the *corrTh* from 0.30 to 0.90 with a step size of 0.10. The *corrTh* is explained in the section 2.3.1 while describing the network construction steps.

For the purpose of comparing our results we perform the same classification experiments using some standard graph features computed on the brain functional connectivity networks. The features are computed using the Brain Connectivity Toolbox (BCT) Rubinov and Sporns (2010), which contains a large selection of complex network measures commonly used for characterizing structural and functional brain connectivity data sets. The features we used are the degree, the topological overlap, the clustering coefficient, the local efficiency and the rich club coefficient. Following are the brief descriptions about the network features used:

- Degree of a node is the number of nodes in the network it is connected to by some edges.
- The *m_th_* step generalized topological overlap measure quantifies the extent to which a pair of nodes have similar *m_th_* step neighbors. Where *m_th_* step neighbors are nodes that are reachable by a path of at most length *m*. We got best results for *m* = 5.
- Clustering coefficient is the fraction of triangles around a node. In other words, it is the ratio of the neighbor nodes count which are connected to each other to the total number of neighbor nodes of the node.
- Local efficiency is the global efficiency computed on node neighborhoods. Where global efficiency is the average of inverse shortest path lengths in the network.
- Rich club coefficient at level *k* is the fraction of edges that connect nodes of degree *k* or higher out of the maximum number of edges that such nodes might share. We compute the coefficients for all the *k* values where 0 ≤ *k* ≤ *K*. Here, *k* is an integer and *K* is the maximum degree found for any node of the training data.

Since each of the network features returns a feature vector whose size depends on the node count of the network, we had to make the node counts same for all the subjects to make the feature sizes same. For this reason we construct the networks in a little different way. Instead of using one power threshold value for selecting highly active voxels for the whole brain, we use separate power thresholds for each of the ROIs of CC200 map. For each of the ROIs, we select the voxels ranked 98 percentile or higher based on their power values. The rest of the network construction process is same as before. The experiments are also set up in the similar fashion as described for our proposed method.

To better understand the physical interpretations of each of the dimensions of the MDS projected space, we performed some analysis. First we compute some global feature values for each of the networks of the KKI training set. A brief description of the computed features is as follows:

- **Density:** it is the fraction of present connection to all possible connection of the network.
- **Global efficiency:** it is the average inverse shortest path lengths of the network.
- **Rich club coefficient:** as described in section 3. The correlation values reported with *x* coordinates of the male and female groups are achieved when *k* = 11 and *k* = 1 respectively.
- **High power node fraction:** it is the fraction of the nodes with power greater than a threshold to the total number of nodes of the network. The correlation value reported with *x* coordinates of female group is achieved when *powTH* = 0.85.

For each of the computed global features, two separate feature vectors are formed for the male and female group of subjects. Please note here each feature vector represents a group of subjects (for e.g., the male and female groups) but not the individual subjects. Then the correlations of the feature vectors are computed with the *x* and *y* coordinates of the 2 dimensional space where networks are projected using the MDS method.

To show the importance of the high power voxel selection step we perform a set of experiments using our method but without the voxel selection step. Finally, we experimentally validate the effectiveness of the node attribute set used in out method. For this purpose, we compute the inter-graph distances using different subsets of the attribute set used. For each of the subsets, inter-graph distances are computed separately followed by the projection of the subjects to a low dimensional space using MDS and classification using SVM. It is not possible for us to compute results for all the possible subsets as there can be 31 different subsets for 5 attributes. Instead we start with one attribute and keep on adding attributes in the subsets. The results show that the classification accuracies steadily increase as we kept on adding attributes in the subset. Finally, we validate on all combinations of 4 attributes to show that even missing one of the attributes of our attribute set decreases the classification accuracy.

## 3. Results

**Figure 2:**
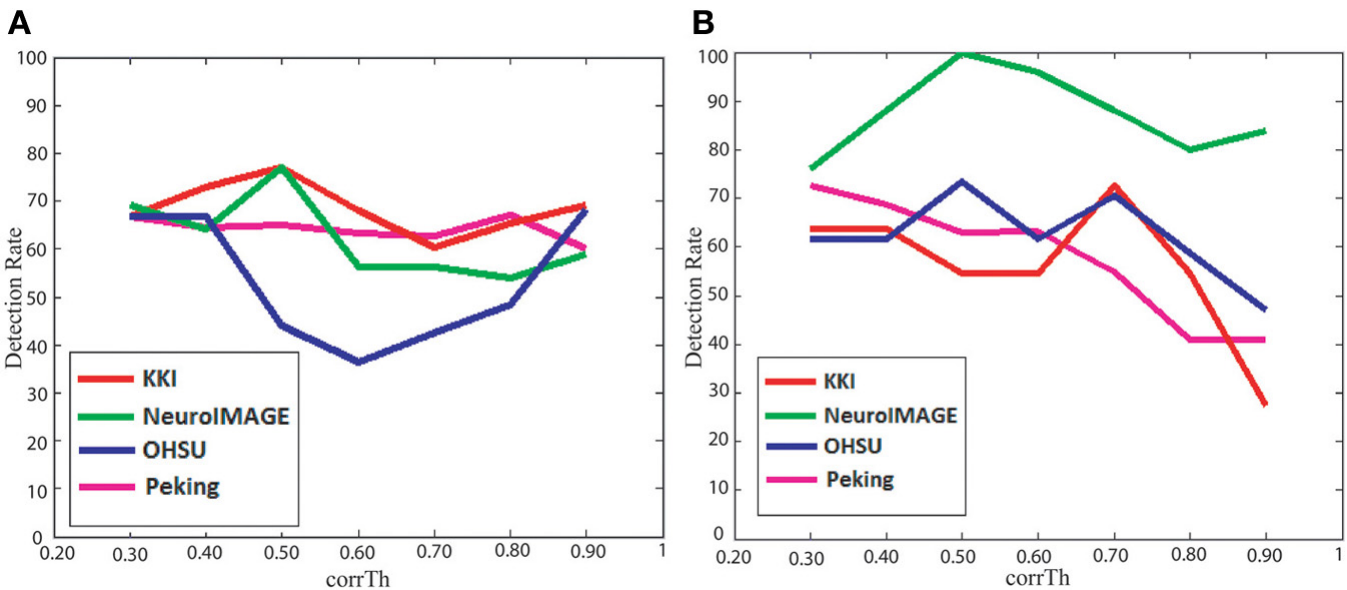
**Figure plots *corrTh* vs. detection rates of our method on the (A) training data sets and (B) test data sets**.

The detection rates of our method, when classification is performed separately on the male and female subjects, are plotted in Figure 2. The plots show how the detection rates vary for the different data centers and with respect to different *corrTh* values. In Table 3 we reported the best detection rates of our method along with the specificity and sensitivity values for all the training sets. The *corrTh* values corresponding to the best detection rates on the training sets are selected and used to get the detection rates for the test centers. One interesting fact is that in most of the cases we get better classification accuracies when experiments are performed on the male and female subjects separately. We achieve an average detection rate of 64.48% on the training data sets and an average detection rate of 62.81% on the test data sets when classification is performed on all the subjects and 70.49% on the training data sets and an average detection rate of 73.55% on the test data sets when classification is performed separately on the male and female subjects.

**Table 3:** **Summary of the results: table shows the best detection rates achieved (along with their specificities and sensitivities) on all the training sets using the proposed method**.

**All subjects**							
**Data centers**	**Training data sets**			**Test data sets**			***corrTh***
	**Detection rate**	**Specificity**	**Sensitivity**	**Detection rate**	**Specificity**	**Sensitivity**
KKI	75.64	100	9.52	54.55	62.50	33.33	0.8
NeuroIMAGE	64.10	68.18	58.82	48.00	64.29	27.27	0.5
OHSU	60.61	65.79	53.53	82.35	89.29	50.00	0.9
Peking	61.20	86.61	21.13	58.82	92.59	20.83	0.6
Average	64.48	84.71	30.66	62.81	83.12	27.27	
**Male female separate**							
KKI	76.92	90.48	36.84	54.55	62.50	33.33	0.5
NeuroIMAGE	76.92	81.82	70.59	100	100	100	0.5
OHSU	68.18	78.95	53.57	61.76	60.71	66.67	0.3
Peking	67.21	83.93	40.85	72.55	74.07	70.83	0.3
Average	70.49	84.53	46.72	73.55	72.73	75.00	

The *corrTh* values are selected from the training sets where we achieve best detection rates. The rates on the test sets for the corresponding *corrTh* values are reported. The values under the heading “Male Female Separate” are computed by averaging the accuracies on the male and female groups.

**Figure 3:**
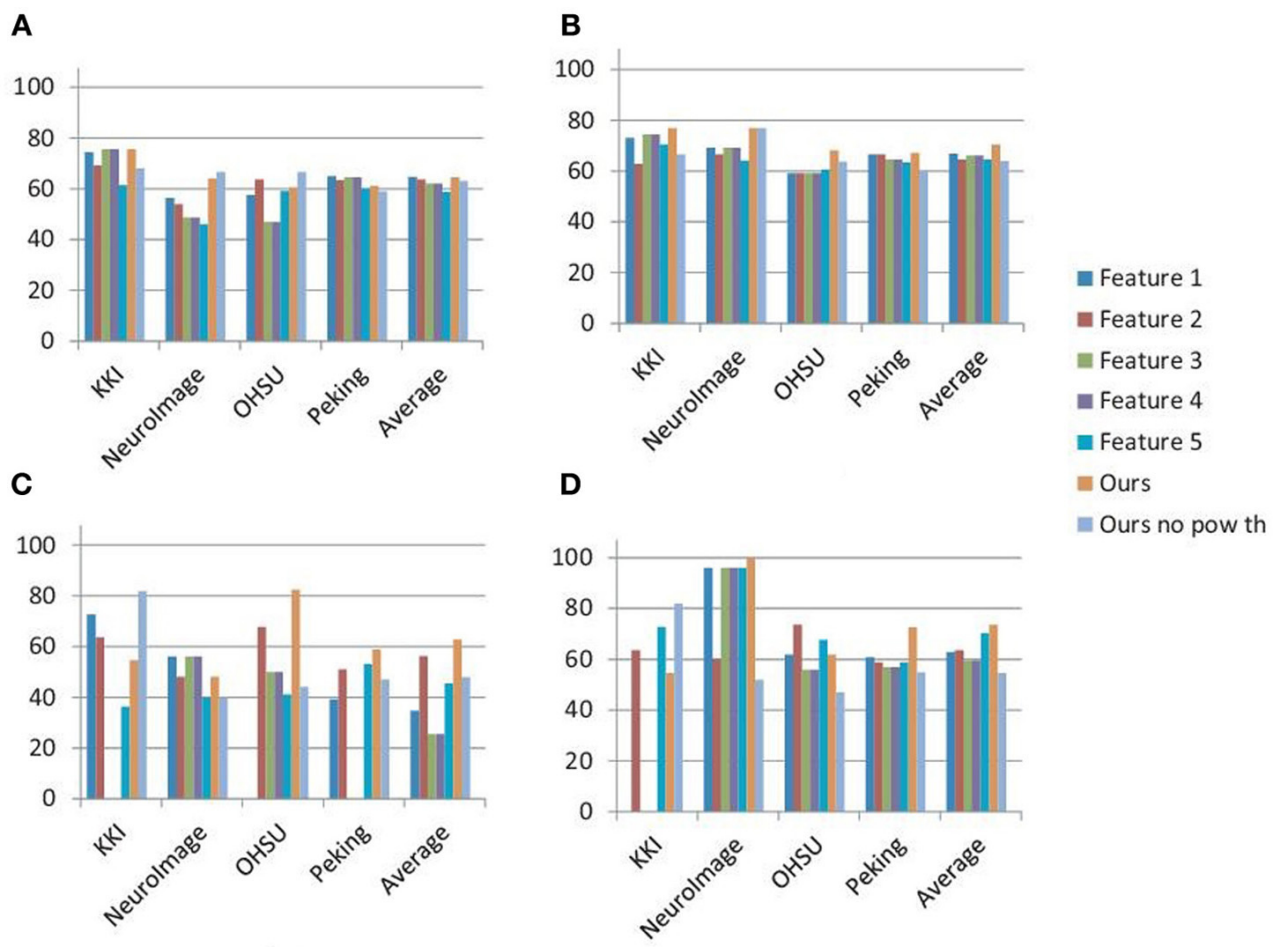
**Summary of the results: figure plots the best detection rates achieved on all the training and test sets using five commonly used network features implemented in the BCT, our method and our method without the high power voxel selection step.** Features 1–5 are the degree, topological overlap, clustering coefficient, local efficiency and rich club coefficient respectively. **(A,B)** Show the results on the training sets when the classification is performed on all the subjects and on the male and female subjects separately. **(C,D)** Show the similar results on test sets. The detection rates of **(B,D)** are computed by averaging the detection rates on the male and female groups.

The detection rates of the classification experiments performed using the standard network features are shown in Figure 3 along with the results of our method. The results are reported separately for each of the data canters as well as the average detection rates are mentioned. It can be seen in almost all of the cases our method performs better than the network features. Also, in average, none of the features performs better than our method when used separately on the male and female subjects. This justifies the need of a specialized method for the analysis of the brain functional problems like ADHD. Please note that we ignored the classification results if any of the specificity or sensitivity is zero. This implies that either all the subjects are classified as ADHD or control. This is why for some of the network features the detection accuracies are zero in Figure 3. Figure 3 also shows the best detection rates of our method when no power threshold is applied for the voxel selection during the network construction step. The lower detection accuracies of these experiments compared to our results justify the importance of the voxel selection step.

**Figure 4:**
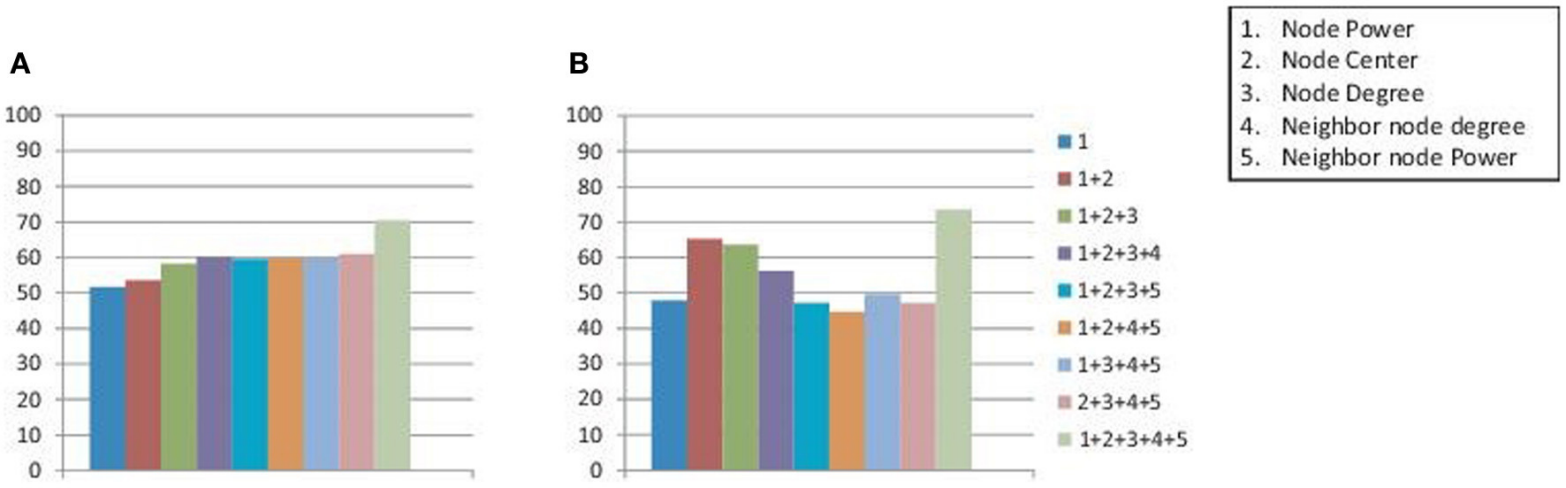
**Figure plots the average detection accuracies on all the data centers when inter-graph distances are calculated using different subsets of node attributes.** The classification is performed on the male and female groups of subjects separately to achieve the reported results on **(A)** training data sets and **(B)** test data sets.

Figure 4 reports the results when different subsets of node attributes are used for the calculation of inter-graph distances. For each of the subsets the average classification accuracies on all the data centers are plotted in the Figure. The results reported are achieved when classification is performed separately on the male and female subject groups. As it can be seen, the best detection rates are achieved when we use all the attributes in the set. This justifies the importance of using all the attributes in the set for inter-graph distance calculation.

## 4. Discussion

In this work we propose a novel framework for automatic detection of the ADHD subjects using the rs-fMRI data of brain. For this purpose we construct the functional connectivity network of the brain and represented it as attributed graph. The first step of the network construction method is the efficient selection of the voxels which will be best to capture the functional activities of the brains with the minimum redundancy. We select the highly active voxels for the construction of the networks where voxel activity levels are measured based on the power of their fMRI time series. Often signal to noise ratio of low active voxel time series are very high. Also, these noisy time series can have considerable correlations with each other which lead to the adding of spurious edges or changing the edge weights of the networks. The intuition behind selection of the highly active voxels is to reduce this noise which can affect the correlation weights of the network edges. As shown in the plots of Figures 3A,B, the voxel selection process in general helps to improve the classification scores. But, we have not experimentally verified what is the ideal power threshold value for this. We used a functional ROI map (CC200) to construct the nodes by clustering the selected voxels which belong to the same ROIs. The active voxel selection step along with the use of CC200 map helped us to reduce the computational cost of our algorithm by a great deal. Compared to around 28000 voxels per brain volume, the average node count of the constructed networks is around 60.

Next, we model the networks as attributed graphs where each node of the networks has its signature. These signatures of the nodes contain information about the local structures of the networks. Then, at the time of inter-graph distance computation step, the Munkres algorithm is used to match these local descriptors in a globally optimized fashion. To discourage the algorithm from matching two nodes which are far apart in the physical space, we uses the Euclidian distance of their coordinates as a parameter of the matching cost computation.

**Figure 5:**
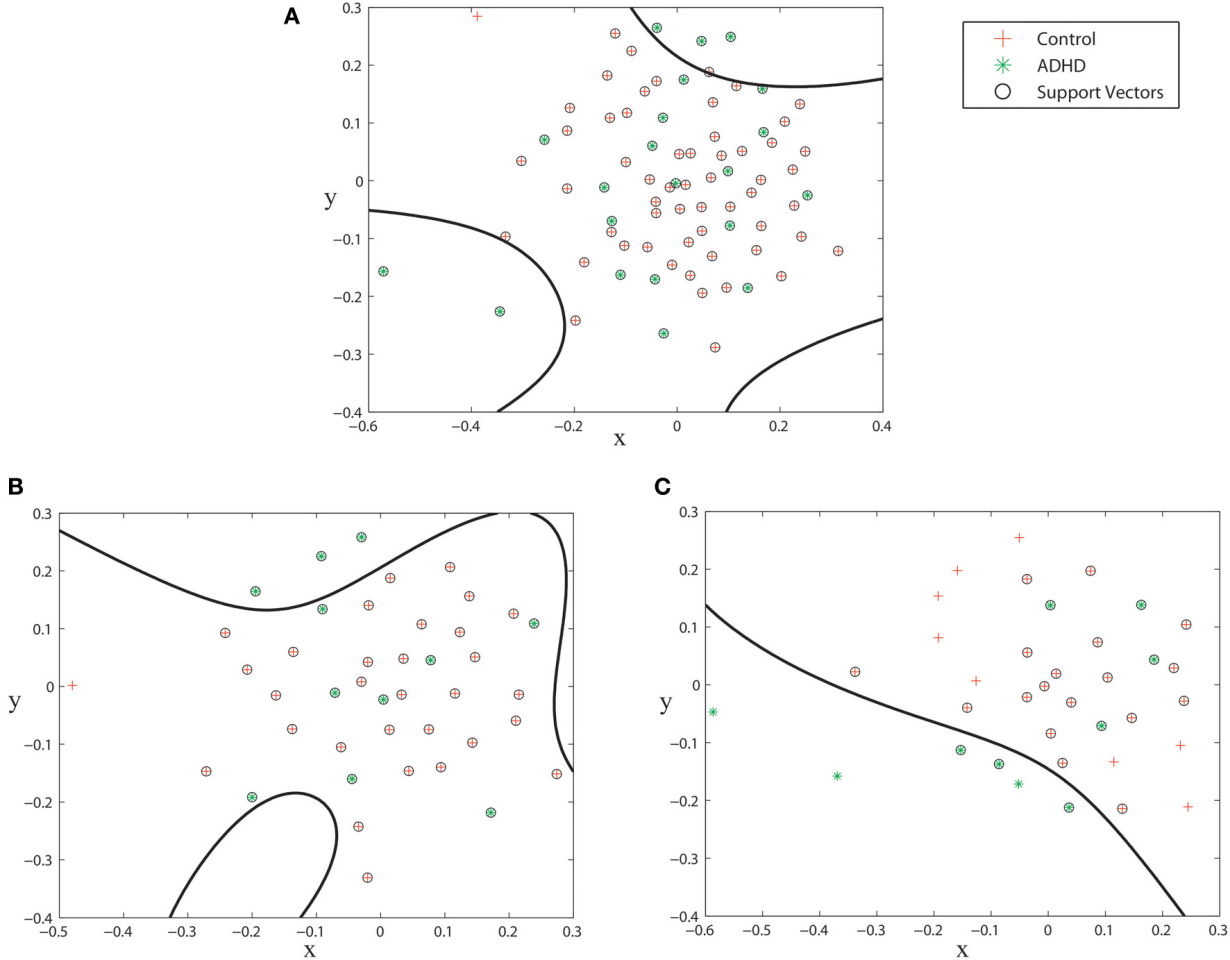
**Subjects from KKI training set plotted on the MDS projected space. (A)** all subjects, **(B)** subjects of the male group, **(C)** subjects of the female group. The spaces are segmented during the SVM training phase.

The inter-graph distance measures allow us to use the MDS technique to map the networks from an unknown space to a 2 dimensional projected space. Figure 5 shows the spacial configuration of the subjects of the KKI training set when mapped to their projected space. As it can be seen, the ADHD subjects can be better segmented when the male and female groups are plotted separately compared to when all the subjects are plotted together. This fact is reflected in the experimental validations where we consistently get better results when classification is done separately on the male and female groups.

We perform an analysis to understand the physical interpretation of the different dimensions of the MDS projected space. For this purpose we computed the correlations of the different global features of the networks with their coordinates in the projected space. The correlation values are reported in Table 4. As it can be seen, the *x* coordinates of the projected spaces of the male and female groups are highly correlated with the density and rich club coefficient features and moderately correlated with the global efficiency. It should be noted that these three features capture different aspect of network edge structures. The last feature shows some correlation with the *y* coordinate of female group.

**Table 4:** **Correlations of the global features of the networks with the *x* and *y* dimensions of the projected spaces of the male and female groups**.

**Global features**	***x*_male_**	***y*_male_**	***x*_female_**	***y*_female_**
Density	0.6906	0.3248	0.8310	0.1070
Global efficiency	0.4594	0.1924	0.5391	0.2578
Rich club coefficient	0.6367	0.4228	0.6482	0.4146
High power node fraction	0.3055	0.1984	0.1338	0.4942

To justify the importance of a specialized method for analysis of the ADHD, we compared our results with some of the standard brain connectivity measures heavily used for functional analysis of the brain. As shown in Figure 3 our method out performs the standard network features by a large margin. Only the topological overlap feature performs similar to our method on the training data sets.

Figure 2 shows how detection rates vary with different correlation thresholds used for the network computation. It can be seen that the peaks of the detection rates are not same for the different data centers. There are two main potential reasons for this variation. First, there are variations in experimental protocols followed by the different data centers. Also, to capture the data different data centers used different scanner models and scanning parameters. Second, the subjects, participated in the different centers, have different age distributions. Mehnert et al. (2013) found changes of functional connectivity measures with age in human brain. The variation of detection rate patterns across the centers indicates that there is a need to follow a more standardize experimental procedure for the future studies.

To conclude, we develop a novel classification framework which is modeled in a computationally efficient fashion as we are able to drastically reduce the functional connectivity networks sizes by efficiently selecting voxels and clustering them. Also, our approach is able to produces impressive classification accuracies (70.49% on training data sets and 73.55% on test data sets) especially on the test sets where we get the better detection accuracies than any of the previously reported results (69.59% by Dey et al., 2012 was the previous best). For this purpose we construct the functional connectivity networks of the brains and use their inter-network distance measures to project them onto a 2 dimensional space. We provide physical interpretations of the dimensions of the projected space in our analysis. Also, we show the superior performance of our method over the standard network measures.

### Conflict of interest statement

The authors declare that the research was conducted in the absence of any commercial or financial relationships that could be construed as a potential conflict of interest.
